# Challenges and Innovations in Alveolar Bone Regeneration: A Narrative Review on Materials, Techniques, Clinical Outcomes, and Future Directions

**DOI:** 10.3390/medicina61010020

**Published:** 2024-12-27

**Authors:** Diana Marian, Giuseppe Toro, Giovanbattista D’Amico, Maria Consiglia Trotta, Michele D’Amico, Alexandru Petre, Ioana Lile, Anca Hermenean, Anca Fratila

**Affiliations:** 1 Department of Dentistry, Faculty of Dentistry, “Vasile Goldiș” Western University of Arad, 94-96 Revolutiei Blvd., 310025 Arad, Romania; marian.diana@uvvg.ro; 2 Multidisciplinary Department of Medical, Surgical and Dental Sciences, University of Campania “Luigi Vanvitelli”, 80138 Naples, Italy; giuseppe.toro@unicampania.it; 3 School of Geriatrics, University of Studies of L’Aquila, 67010 L’Aquila, Italy; giovanbattista.damico.dott@outlook.it; 4 Department of Experimental Medicine, University of Campania “Luigi Vanvitelli”, 80138 Naples, Italy; mariaconsiglia.trotta2@unicampania.it (M.C.T.); michele.damico@unicampania.it (M.D.); 5 Department of Prosthodontics, “Carol Davila” University of Medicine and Pharmacy, 050474 Bucharest, Romania; 6 “Aurel Ardelean” Institute of Life Sciences, “Vasile Goldiș” Western University of Arad, 310025 Arad, Romania; hermenean.anca@uvvg.ro; 7 Department of Dental Medicine and Nursing, Faculty of Medicine, “Lucian Blaga” University of Sibiu, 550169 Sibiu, Romania; anca.fratila@ulbsibiu.ro; 8 Military Clinical Emergency Hospital of Sibiu, 550024 Sibiu, Romania

**Keywords:** alveolar bone regeneration, biomaterials, guided bone regeneration

## Abstract

This review explores the recent advancements and ongoing challenges in regenerating alveolar bone, which is essential for dental implants and periodontal health. It examines traditional techniques like guided bone regeneration and bone grafting, alongside newer methods such as stem cell therapy, gene therapy, and 3D bioprinting. Each approach is considered for its strengths in supporting bone growth and integration, especially in cases where complex bone defects make regeneration difficult. This review also looks at different biomaterials, from bioactive scaffolds to nanomaterials, assessing how well they encourage cell growth and healing. Personalized treatments, like customized 3D-printed scaffolds, show promise in enhancing bone formation and tissue compatibility. Additionally, signaling molecules, like bone morphogenetic proteins, play a crucial role in guiding the process of bone formation and remodeling. Despite these advances, challenges remain—particularly with severe bone loss and with refining biomaterials for more reliable, long-term outcomes. This review proposes combining advanced materials, regenerative technologies, and personalized approaches to achieve more effective and consistent outcomes in oral and maxillofacial surgery.

## 1. Introduction

The alveolar bone is a critical component of the tooth-supporting structure, consisting of the alveolar bone proper, cortical bone, alveolar crest, and trabecular bone. Its preservation is essential for the success of implant-supported restorations. This bone develops alongside the teeth and undergoes resorption following tooth extraction [1]. After an extraction, maintaining the alveolar crest is crucial to achieving good aesthetic outcomes with dental implants [2]. The alveolar bone distributes the forces from chewing to surrounding structures, adapting to functional changes over time [3]. The periodontium, which includes the alveolar bone, periodontal ligament, cementum, and gingiva, supports the teeth structurally, absorbs biting forces, and protects the surrounding tissues [3]. Understanding the microarchitecture of this bone is vital for improving the success of implant therapy [4].

In cases of bone loss, the alveolar ridge can be augmented horizontally or vertically using membranes that create the optimal conditions for bone regeneration [5]. The success in these procedures relies on proper management of the surgical flap, stabilization of the graft and membrane, and a tension-free closure. For severe bone resorption, regenerative techniques using stem cells, like mesenchymal stem cells (MSCs) and endothelial progenitor cells (EPCs), show great potential in periodontology, maxillofacial surgery, and implantology [6,7].

Bone regeneration is a highly intricate process that depends on the interaction between cells and biomaterials to produce new bone tissue. To enhance this process, a number of approaches have been explored, such as using synthetic materials, scaffolds with bioactive molecules, nanotechnology, biomimetic designs, and therapies based on living cells [8]. Research data have shown that using bone morphogenetic proteins (BMPs) within collagen scaffolds or blood clots can significantly boost bone healing and promote the new bone tissue formation [9]. Additionally, recent studies highlight the crucial role that inflammation and cellular signaling pathways play in the bone repair process [10]. A novel and promising approach focuses on using the body’s own tissues, especially the periosteum, to act as a bioreactor that aids in bone regeneration [11].

This review explores key strategies and challenges in alveolar bone regeneration, highlighting biological processes, materials, and techniques, including guided bone regeneration, bone grafting, and the use of growth factors and stem cell therapies. It examines the effectiveness and potential limitations of these methods, with the goal of improving outcomes in dental implants and periodontal treatments.

The articles and research papers included in this review encompass the latest advancements and relevant studies in alveolar bone regeneration up to 2024. A thorough literature search was conducted using reputable databases such as PubMed, Scopus, and Web of Science to ensure comprehensive coverage of peer-reviewed and reliable sources.

In conducting the literature review, we utilized a comprehensive set of keywords to ensure the retrieval of relevant and high-quality studies. The key terms included: alveolar bone regeneration, biomaterials for bone regeneration, guided bone regeneration, bone grafting techniques, stem cell therapy in alveolar bone, 3D bioprinting for bone regeneration, bone morphogenetic proteins (BMPs), innovative approaches in oral implantology, horizontal and vertical bone augmentation, challenges in bone regeneration.

## 2. Bone Biology

Bone is a mineralized tissue that constantly undergoes changes—through remodeling, resorption, and deposition—thanks to the activity of specialized cells. Though it provides structure and rigidity, bone is a living, dynamic tissue capable of both healing and growth. It interacts with materials like titanium implants, helping with osseointegration, which is key in rehabilitation. In addition to mechanical functions, bone also serves as a reservoir for inorganic ions and provides attachment points for muscles, tendons, and ligaments. Additionally, it holds stem cell niches, which are essential for tissue repair [12,13].

Bone biology encloses three key types of cells: osteoblasts, osteoclasts, and osteocytes, which work together to form, support, and remodel bone tissue [14,15]. These cells are tightly regulated by signaling molecules, playing vital roles in the development and maintenance of bone throughout life [15]. Understanding how these cells function is critical for studying bone health and diseases that may affect the skeletal system [16]. Bone behavior is also influenced by evolutionary factors and is closely linked to other organs and systems in the body [17]. In short, bone provides structural support, facilitates movement by anchoring muscles, and plays a vital role in mineral storage and blood cell formation. Understanding bone biology and key cell signaling pathways is essential for studying skeletal metabolism and related disorders.

Bone tissue contains various growth factors, including bone morphogenetic proteins (BMPs), transforming growth factor-beta (TGF-β), insulin-like growth factors (IGF-I and IGF-II), platelet-derived growth factor (PDGF), and fibroblast growth factors (bFGF and aFGF) [18]. The interaction between hormones, growth factors, and cytokines is crucial for bone and cartilage development [19]. These growth factors play a critical role in bone grafting and fracture healing, promoting various processes such as cell growth, differentiation, and repair [20]. Osteoblasts, the bone-forming cells, produce these growth factors, which help in bone repair through autocrine and paracrine actions [21]. Key factors include:

- Bone morphogenetic proteins (BMPs) are essential for the differentiation of mesenchymal stem cells into osteoblasts and are crucial for bone formation and repair.
- Transforming growth factor-beta (TGF-β) promotes osteoblast proliferation and differentiation while regulating osteoclast activity through the RANKL/OPG pathway.
- Insulin-like growth factors (IGFs) like IGF-I and IGF-II are critical for bone growth, encouraging osteoblast proliferation and protein synthesis within the bone matrix.

Bone health and development depend on several key signaling pathways, such as Wnt, Hedgehog (HH), PTHrP, TGF-β, NOTCH, FGF, and BMP. These pathways, along with transcription factors like RUNX2 and SOX9, help regulate bone cell formation and maintenance [22,23]. Disruptions in these systems can lead to bone disorders.

The most important signaling pathways involved in other physiological activities of the bone tissue include Wnt/β-Catenin, important for osteoblast function and bone formation; RANK/RANKL/OPG, which balances the activity of osteoclasts to control bone resorption; and Notch, which guides the differentiation of bone cells, crucial for bone remodeling. Understanding these pathways is essential for advancing treatments for bone-related conditions, like osteoporosis or the inability to restore bone tissue in large defects [24].

**Figure 1:**
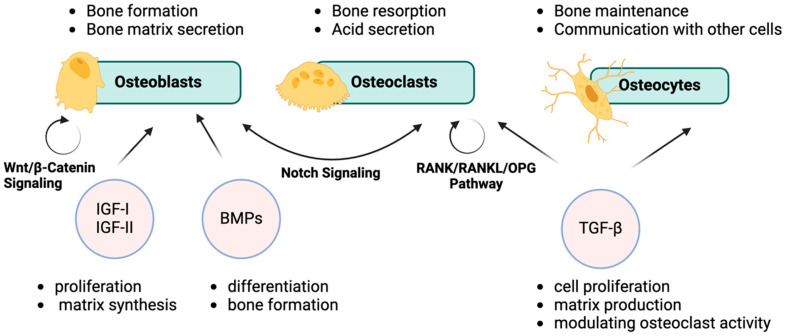
Key signaling pathways and cellular interactions in bone homeostasis. This figure was created with BioRender.com.

Figure 1 illustrates the key signaling pathways, such as Wnt/β-Catenin, Notch, and RANK/RANKL/OPG, and their roles in regulating the functions of osteoblasts, osteoclasts, and osteocytes during bone homeostasis and remodeling.

## 3. The Role of the Periodontal Ligament (PDL) in Alveolar Bone Homeostasis and Regeneration

The periodontal ligament (PDL) is a key tissue that connects your teeth to your jawbone, ensuring their stability and health. It plays a crucial role in orthodontic treatments, as it facilitates tooth movement by responding to the pressure applied. The PDL senses these forces and initiates remodeling processes that adjust both the ligament and the bone around your teeth. It is made mostly of collagen fibers, which give it the strength to handle chewing and movement [25]. It also has cells like fibroblasts, cementoblasts, and osteoblasts, which help keep the PDL healthy and repair it when needed. Moreover, the presence of stem cells in the PDL offers significant potential for it to regenerate damaged tissues [26]. In summary, the PDL is critical for holding our teeth in place, helping them move when necessary, and healing them when needed.

In addition to anchoring teeth, the PDL plays a big role in maintaining the health of the surrounding bone and gums. It contains cells that help repair and regenerate the tissues around your teeth by producing materials that support the nearby bone and gums [27]. The PDL constantly adapts to the stress of chewing or tooth movement by remodeling itself, making it an important part of the healing process for your gums and bone [28]. It also regulates calcification, promotes the formation of bone and cementum, and maintains the space around your teeth, acting like a protector for the whole tooth-supporting system [29]. What is even more impressive is the PDL’s ability to regenerate. The cells in the PDL can turn into the types of cells needed to repair the bone and gums around your teeth, which helps in healing and regrowth [30]. Research has shown that stem cells from the PDL can be used to help restore damaged periodontal tissues [31]. New technologies, like special scaffolds and membranes, are even being developed to make the regeneration of the PDL and surrounding bone even better, showing just how important the PDL is in modern tissue repair and future medical treatments [32].

## 4. Methods of Alveolar Bone Regeneration

### 4.1. Guided Bone Regeneration (GBR)

Guided bone regeneration (GBR) is a surgical technique commonly used in dentistry and maxillofacial surgery to restore lost bone. Its primary goal is to promote bone growth by excluding non-osteogenic tissues, such as epithelium, from infiltrating the defect area. GBR is particularly beneficial for preparing sites for dental implants or repairing bone defects caused by trauma, tooth extraction, periodontal disease, or congenital conditions [33]. The core principle of GBR involves using a barrier membrane to separate the bone defect from surrounding soft tissues [34], ensuring that only bone cells populate the defect site, thus encouraging new bone formation [35].

Non-resorbable membranes, such as expanded polytetrafluoroethylene (ePTFE) and dense PTFE (dPTFE) (Table 1), provide strong mechanical stability and maintain the necessary space for bone regeneration [33]. However, they must be surgically removed after serving their purpose. In response to the challenges of large vertical defects, titanium-reinforced membranes were developed to enhance strength and volume stability [27,36,37]. Titanium-reinforced d-PTFE has become a preferred choice for GBR due to its ability to achieve high vertical bone regeneration with a low complication rate [38]. While effective, these membranes require a second surgery for removal, increasing patient discomfort and costs [39].

Absorbable membranes, made from natural materials such as collagen, chitosan, and gelatin, were developed to address the limitations of non-resorbable membranes. These biodegradable materials are gradually replaced by the patient’s tissue during the healing process [40]. Despite their advantages, absorbable membranes can fail in extensive bone lesions due to lower mechanical strength [41].

**Table 1:** Overview of membrane types: advantages, disadvantages, and outcomes.

Membrane Type	Examples	Pros	Cons	Results According to the Literature
Non-resorbable membranes	expanded-PTFE (e-PTFE) (ex. Gore-Tex^®^) high density-PTFE (d-PTFE) (ex. Cytoplast™ Regentex GBR-200 or TXT-200 Titanium mesh	- High mechanical stability - Excellent barrier function - Long-term space maintenance	- Requires a second surgery for removal - Potential for membrane exposure - Rigid; can cause soft tissue separation	- Titanium-reinforced membranes provide the highest vertical bone regeneration with a low complication rate [42] - Effective in complex cases requiring long-term stability [43,44]
Resorbable membranes	Natural			
Collagen	Bio-Gide^®^ Jason ^®^ Ossix^®^	- Biocompatible - Supports osteoblast migration - No need for removal	- Limited mechanical stability - Limited barrier functionality due to their resorption	[45,46,47,48]
Chitosan	Derived from crustacean shells	- Biocompatible - Antibacterial properties - Promotes wound healing	- Limited mechanical strength - Rapid resorption may limit functionality	- Chitosan shows promise in GBR but needs enhancements for mechanical stability and slower degradation [49]
Gelatin	Derived from collagen	- Biocompatible - Easy to handle - Supports cell adhesion	- Poor mechanical stability - Rapid resorption	- Gelatin is useful in tissue engineering but requires crosslinking or reinforcement for improved stability [50]
Resorbable membranes	Synthetic			
Polylactic acid (PLA)	PLA-based membranes	- Customizable degradation rates - Stronger than natural materials	- Potential for acidic degradation products - May still lack long-term stability	- PLA membranes can be tailored for specific uses but require careful control of degradation byproducts [51]
Polycaprolactone (PCL)	PCL-based membranes	- Long degradation time - Good mechanical strength	- Slower resorption rate - Potentially less biocompatible than natural options	- PCL is suitable for long-term applications but may need combination with other materials for enhanced biocompatibility [52]
Combinations				
Collagen + silk	Hybrid membranes	- Enhanced mechanical strength - Improved cell adhesion and proliferation	- Complexity in fabrication - Possible variability in degradation rates	- Collagen–silk composites show improved mechanical properties and support osteogenesis better than collagen alone [53]
Gelatin + chitosan	Composite membranes	- Dual-layer GBR membrane - Offers superior barrier function	- Complex fabrication process	[54]
Chitosan + graphene	Composite membranes	- Increased mechanical strength - Enhanced electrical conductivity - Antibacterial properties	- Complex fabrication process	- Chitosan–graphene composites provide enhanced mechanical stability and bioactivity, making them suitable for bone regeneration [55]
Graphene oxide/chitosan/hydroxyapatite	Composite membranes	- Improved mechanical strength and surface hydrophilicity - Enhanced osteoblast adhesion, differentiation, and mineralization	- Complexity in manufacturing - Potential issues with uniform integration	- This combination improves bone regeneration by enhancing the mechanical and bioactive properties of the membranes [56]
Collagen + hydroxyapatite	Biomimetic composites	- Mimics natural bone matrix - Promotes osteointegration - Stronger and more stable	- Potential for inconsistent integration - Higher cost and complexity	- Collagen–hydroxyapatite membranes combine the benefits of both materials, improving bone regeneration outcomes [57]
PLA + bioactive glass	Composite membranes	- Enhanced bioactivity - Controlled degradation - Supports bone regeneration - Induces angiogenesis	- Potential brittleness - Requires careful balancing of properties	- PLA combined with bioactive glass improves bioactivity and supports new bone formation more effectively than PLA alone [58]
Chitosan–collagen–hydroxyapatite membranes	Composite membranes	- Asymmetric structure, biodegradability, biocompatibility, antibacterial activity	- Complexity in design and fabrication - Potential for variable degradation	- Chitosan–collagen–hydroxyapatite membranes provide enhanced bone regeneration capabilities, combining the strengths of each component [59]
Chitosan/fibroin–hydroxyapatite and collagen membrane	Composite membranes	- Improved mechanical properties - Enhanced bioactivity - Supports osteogenesis	- Complex manufacturing - Potential cost issues	- This combination shows promise for enhanced bone regeneration with improved stability and bioactivity [60]

Third-generation membranes for guided bone regeneration (GBR) represent a significant advancement in bone healing [61]. Beyond simply acting as barriers to unwanted tissues, these membranes enhance the healing by slowly releasing substances that boost bone growth. These high-tech membranes are designed to make GBR work more efficiently, helping bones heal faster and connect better with the surrounding tissue. Table 2 provides an overview of these membranes, highlighting their features, mechanisms for releasing bioactive agents, and various applications.

### 4.2. Bone Grafting

Bone grafting is a surgical method used to repair or replace missing bone, and there are different types of grafts available, including autografts (using your own bone), allografts (bone from a donor), and synthetic options.

Autografts are considered the best option because they not only provide a structure for new bone to grow on but also help stimulate bone growth and contain cells that can turn into bone. Since the bone graft is sourced from the patient’s own body, the risk of rejection or infections is significantly reduced. Common donor sites include the hip, shin, and heel [67,68].

Xenografts, typically derived from animal sources such as bovine bone, are another well-studied option. For example, true bone ceramics (TBC) works better for femur defects, while decalcified bone matrix (DBM) is better for radial defects. Xenografts are often used in trauma and orthopedic surgeries, especially in joint fractures or replacement surgeries [69]. They provide good structural support and do not require bone harvesting from the patient, but there is a risk of the body rejecting them, and they do not help grow new bone as well as autografts [70].

Alloplasts, synthetic bone substitutes, are commonly used in dental surgeries. They act as fillers and support structures to encourage bone growth, and they are easy to receive without the risk of disease transmission. However, unlike autografts, alloplasts do not inherently stimulate bone formation, and certain types of synthetic or donor-based grafts carry a minimal risk of disease transfer [71,72].

Table 3 provides a comprehensive overview of the various types of bone grafts, including autografts, allografts, xenografts, and synthetic options, highlighting their advantages and disadvantages.

### 4.3. Bone Morphogenetic Proteins (BMPs)

Bone morphogenetic proteins (BMPs) are essential proteins that help cells grow and develop, especially in bones and cartilage. They play a significant role in skeletal formation and ensuring proper bone development [83]. In addition to helping with bone growth, BMPs are also connected to cancer, affecting how the disease spreads and how the immune system responds to it [84,85]. In fact, certain BMPs are being studied for their potential to predict the spread of breast cancer to lymph nodes [86].

In bone healing, BMPs like BMP-2, BMP-4, BMP-6, BMP-7, and BMP-14 are highly effective in initiating bone formation and establishing the structural framework for new bone [87,88]. These proteins are widely used in many medical treatments, including spinal fusion surgeries [89], or treating open bone fractures [90]. However, using BMPs in large amounts can cause side effects, prompting ongoing research to determine optimal dosages and how to control their release safely [91]. Despite these challenges, BMPs—especially BMP-2—are widely used in dental procedures like regenerating jawbone and supporting dental implants.

Table 4 highlights how BMPs are used in jawbone regeneration, highlighting their benefits and challenges.

### 4.4. Stem Cell Therapy

Stem cell therapy is showing great potential for dental regeneration [92]. Mesenchymal stem cells (MSCs) are particularly promising due to their ability to differentiate into bone-forming osteoblasts or tooth-forming odontoblasts, as well as their role in modulating immune responses. This versatility makes them a very promising option for regenerating both bone and teeth [93,94,95]. MSCs can be sourced from many different tissues, including both neonatal and adult sources, making them relatively easy to access for regenerative treatments [96,97].

Bone marrow mesenchymal stem cells (BMMSCs) are particularly well studied for their role in bone regeneration. They contribute to bone formation and regulate osteoclast activity, which is essential for maintaining bone balance. BMMSC dysfunction has been linked to bone disorders like osteoporosis, but their anti-inflammatory properties also make them a promising option for treating bone defects and conditions like osteopenia through cytotherapy and tissue engineering approaches [17,98,99].

Adipose-derived mesenchymal stem cells (ADMSCs) offer a great alternative to BMMSCs because they are easier to access and available in larger quantities [100,101]. They also remain functional in less-than-ideal conditions, making them highly suitable for bone repair, treating osteopenia, and for use in engineered bone grafts [102,103].

Dental stem cells (DSCs), including dental pulp stem cells (DPSCs), are also incredibly useful due to their easy accessibility, abundant supply, and the fact that collecting them involves minimal discomfort for the donor. This makes them a great option for regenerating tissues like dental pulp, periodontal ligaments (PDL), and even for developing biological tooth implants [104]. DPSCs are particularly great for restoring dentin (the layer underneath your enamel), and they even have neurovascular properties, making them ideal for regenerating both bone and teeth [105,106].

Periodontal ligament stem cells (PDLSCs), derived from the periodontal ligament (PDL) tissue, are another promising option for regenerating periodontal tissues. These stem cells express markers for both cementoblasts and osteoblasts, helping to form cementum (the layer covering tooth roots) and bone-like structures. PDLSCs can be sourced from different dental tissues, giving them a lot of flexibility for use in periodontal regeneration [107,108].

Stem cells from human exfoliated deciduous teeth (SHED) play a crucial role in pulp regeneration. They show enhanced potential for turning into tooth-forming cells (odontogenic) and bone-forming cells (osteogenic), making them a valuable resource for both bone and tooth regeneration—even after being cryopreserved [109,110].

Stem cell-based regenerative strategies for bone and dental tissue are summarized in Table 5.

## 5. Clinical Indications and Implications

Bone augmentation techniques are a critical part of dental implant therapy. They help ensure stable implants and more predictable results. One of the common methods used is guided bone regeneration (GBR) with barrier membranes, which has been a standard practice for a long time [117,118]. Procedures like ridge preservation and bone grafting are necessary when bone loss occurs due to various factors [5]. However, these procedures often involve higher costs, risks of complications, and longer treatment times, since both hard and soft tissue augmentation are often needed to meet functional and aesthetic goals [119]. Even patients with health conditions like acromegaly can benefit from bone augmentation, especially when autologous bone is not an option [120].

For dental implants to be successful, there must be enough bone width, height, and vitality, so choosing the right materials for bone reconstruction is crucial [121]. Ridge augmentation procedures, for instance, help to increase the width of the alveolar ridge, making it easier to place implants [122]. In aesthetic zones, immediate implant placement and provisionalization (IIPP) are popular approaches but carry risks such as labial bone perforation. To mitigate this, some surgeons combine root resection with GBR, which helps lower the chances of bone perforation [123].

In the posterior mandible, both simultaneous and delayed implant placement after following lateral ridge expansion show similar results in terms of bone loss and buccal thickness changes [124,125].

When it comes to repairing the alveolar ridge after tooth extraction or trauma, several methods can be used. These include calcined cattle bone grafts or tissue-engineered constructs with rabbit bone marrow mesenchymal stem cells and beta-tricalcium phosphate [126]. Additionally, autotransplantation or replantation of extracted teeth combined with artificial bone grafting has proven effective in repairing alveolar bone defects [127,128,129]. These methods aim to preserve the alveolar ridge and prepare it for implant placement, minimizing the need for additional treatments.

Treating periodontal bone defects and furcation involvement can be challenging, especially in advanced cases. Non-surgical cleaning may not be enough, and surgery might be needed for root debridement and bone regeneration. New treatments, like using injectable platelet-rich fibrin (i-PRF) with demineralized freeze-dried bone allograft (DFDBA) for Class II furcation defects, show promising results [130,131].

In some cases, a sinus lift or augmentation is required for placing implants in the upper back jaw (posterior maxilla), where there might not be enough bone. Common techniques for this include the lateral window or transalveolar approaches. Autografts (using the patient’s own bone) are considered the best option, but alternatives like allografts, xenografts, and bone morphogenetic proteins can also be effective [132,133,134,135]. Studies comparing the lateral and transcrestal approaches for maxillary sinus augmentation show that both methods are effective and produce similar amounts of bone gain [136,137].

## 6. Challenges in Alveolar Bone Regeneration

Alveolar bone regeneration presents significant challenges, particularly when dealing with horizontal bone loss, which is a common issue in periodontitis [138]. Unfortunately, this type of defect is particularly difficult to predict in terms of regeneration, as current procedures are not perfect [138].

Traditional non-absorbable membranes used in bone regeneration require a second surgical procedure to remove them, while absorbable membranes often lack sufficient mechanical strength to address larger bone defects [139]. Techniques like socket preservation, which aim to keep the alveolar bone intact after tooth extraction, have shown mixed results when combined with immediate implant placement [140]. Injectable biomaterials offer another option but come with risks like infection, and their application through minimally invasive techniques remains challenging [141]. Selecting the appropriate regenerative methods and biomaterials is crucial for achieving successful outcomes.

Several biological factors can impact how predictable these procedures are. Age, smoking, and overall health play major roles in whether or not alveolar bone regeneration works as intended [138]. With aging, changes in bone metabolism and slower healing times can make regeneration harder [142]. Conditions like diabetes or immune system issues further complicate the process [129], and smoking reduces blood flow and harms the immune system, making bone regeneration and healing slower [143,144].

Despite these challenges, bone augmentation has been successfully performed on patients aged 4 to 68 years old [145,146], with a significant increase in bone width and height and a 79.9% retention rate after six months [147]. Techniques like split bone block methods have also been effective for restoring alveolar bone before placing implants, showing low graft resorption and high success rates. New treatments using romosozumab and MSC-CM are promising in boosting regeneration [148].

When it comes to grafting materials, there are several choices: autografts, allografts, xenografts, and synthetic options. Autografts (taking bone from the patient’s own body) are still considered the best option but require an extra surgery to harvest the bone, and availability is limited [149]. Allografts and xenografts are good at guiding bone growth but come with risks of infection or immune rejection [150]. Synthetic grafts, such as calcium sulfates, tricalcium phosphate, and hydroxyapatite, are available in unlimited amounts and eliminate the risk of disease transmission. However, they may lack sufficient biomechanical strength and are less effective in stimulating bone growth compared to autografts [151].

Infection control is another big challenge in bone banks, where the risk of contamination is high [152,153]. Antibiotic-infused grafts are showing potential, but standardized methods are still needed [154]. Achieving wound closure after grafting is also tricky—primary closure (closing the wound at the time of surgery) seems to result in better bone healing [155], but further improvements in delivery systems for targeted treatments and the biocompatibility of antibacterial agents are needed [156].

Gingival biotype, or the thickness of gum tissue, also plays a role in the final aesthetic outcomes. Thicker tissues are generally more resistant to trauma, making the identification of the biotype before crucial for achieving optimal results [157]. Advanced surgical planning is also key for regenerating papillae (the small triangles of gum tissue between teeth) and ensuring the right emergence profiles for implants, both of which contribute to better overall results [158].

In conclusion, alveolar bone regeneration is a complex process with many influencing factors. Improving biomaterials, grafting techniques, and personalized treatment approaches will be essential for overcoming these challenges and improving patient outcomes. Furthermore, effective oral hygiene practices [159], along with regular follow-ups, can significantly contribute to the overall effectiveness of bone regeneration treatments and promote long-term oral health.

## 7. Emerging Innovations and Technologies

Emerging techniques in alveolar bone regeneration are expanding the possibilities for effective treatments [160]. One promising method is the use of collagenated porcine bone grafts, which closely mimic natural bone. These have shown to be both safe and effective in various regenerative treatments. On the technological front, 3D printing (additive manufacturing) has revolutionized the field by enabling the creation of porous structures ideal for vertical bone growth. These structures support the formation of new blood vessels and enhance the bone regeneration process [161]. Additionally, advancements in stem cell-based therapies, particularly those combining stem cells with bone tissue engineering, are showing great potential. Stem cells derived from dental tissues or reprogrammed as induced pluripotent stem cells have demonstrated the ability to generate new bone, offering a transformative solution for alveolar bone defects [142]. Personalized scaffolding techniques, such as 3D bioprinting, have also made significant strides in creating patient-specific structures that help regenerate both periodontal and alveolar bone [162].

3D printing has completely transformed how we approach bone tissue engineering. With it, we can now design personalized scaffolds that not only encourage bone formation but also support bone graft materials throughout the healing process. Different materials have been used in this process, including magnesium-containing silicate (CSi-Mg), acrylate epoxidized soybean oil (AESO), and polycaprolactone (PCL) [163,164,165]. Research has also explored integrating graphene oxide (GO) with 3D-printed scaffolds to address complex bone defects. Although this shows a lot of potential, there are still regulatory and financial challenges to overcome before these technologies can be widely adopted [166,167].

Nanoparticles are emerging as a transformative tool in bone regeneration, enabling more precise delivery of drugs and growth factors to improve bioavailability and treatment outcomes. These tiny particles come in various forms, like liposomes, micelles, and polymer-based nanoparticles, all designed to enhance drug delivery and improve effectiveness [168,169,170,171,172]. Nanoparticles are already being widely studied for cancer treatments, with some even receiving FDA approval. However, further research is needed to fully understand how they interact with the immune system and to address any potential health risks.

Gene therapy is an increasingly promising approach in bone regeneration, focusing on enhancing the expression of critical bone-growth genes such as TGF-β1 and BMP-2, which are essential for bone formation. Advances in non-viral methods, including modified BMP-2 sequences, are helping to improve the effectiveness of these therapies and prolong growth factor release [173,174,175]. Nonetheless, it holds the promise of revolutionizing bone regeneration by addressing challenges at the genetic level.

Low-level laser therapy (LLLT) is another promising technique in bone regeneration. This technique has been shown to help heal fractures, stimulate blood vessel growth, and even promote the bone-forming abilities of stem cells. It works by activating photochemical pathways that increase energy production (via ATP) and reduce inflammation, all of which help support the formation of bone tissue [176,177,178,179]. However, more research is needed to fine-tune laser protocols and better understand how it supports bone regeneration on a molecular level.

## 8. Future Directions

The future of alveolar bone regeneration is advancing in exciting directions. A big focus is on developing multidisciplinary strategies to better address horizontal bone loss, which is a tough problem to solve. Researchers are exploring innovative biomaterials and therapeutic approaches, both in the lab and in clinical settings, to improve how we regenerate horizontal bone. This is seen as a crucial step forward in advancing regenerative therapies overall [138,180]. One particularly promising development is combining recombinant human bone morphogenetic proteins (rhBMPs) with bone grafts, which is showing great potential for repairing larger defects in the jawbone [143].

Nanotechnology is also shaping the future of bone and periodontal regeneration. For example, calcium phosphate nanoparticles are being studied for use in dental resins to deliver bioactive agents that encourage bone growth and healing around the teeth. Combining advances in nanotech with new biomaterials and clinical methods holds a lot of promise for revolutionizing tissue engineering in the oral and facial regions [181].

Artificial intelligence (AI) and machine learning (ML) are set to significantly impact bone and periodontal regeneration. These technologies are already being used to model how bone grows within scaffolds, which can help optimize treatments [182]. AI and ML are also being used to predict how well bone regeneration might work for specific patients, which can help doctors make better decisions during surgeries and improve patient care. As these technologies improve, it is likely they are expected to deliver even more precise predictions and efficient treatment solutions [183,184].

The emergence of personalized bone regeneration strategies is transforming the field, focusing on treatments tailored to each person’s genetic profile and unique microbiome. This precision medicine approach has the potential to significantly enhance the effectiveness of therapies while minimizing the risk of complications. Researchers are already studying the molecular behavior of mesenchymal stem cells (MSCs) to better understand how they interact with our genes. These insights could unlock entirely new ways of approaching bone regeneration [185,186]. The shift toward personalized treatments is a major area of growth in tissue engineering and bone biology [187,188].

In summary, the future of alveolar bone regeneration looks incredibly promising, with AI, nanotechnology, gene therapy, and personalized medicine all offering groundbreaking ways to treat bone loss and improve outcomes for patients.

This review highlights several limitations. First, the heterogeneity of the included studies and lack of standardized clinical protocols make direct comparisons difficult. While emerging technologies such as 3D printing and nanotechnology show promise, their clinical application is still in early stages, and long-term data are lacking. Additionally, the outcomes of alveolar bone regeneration are influenced by patient-specific factors like age, health conditions, and smoking, which were not deeply explored. Severe bone defects remain challenging to address with current methods.

## 9. Conclusions

Alveolar bone regeneration remains a complex challenge, heavily influenced by factors such as age and overall health. However, recent advancements in technology are opening new possibilities. Innovations in nanotechnology, stem cell research, and growth factors are driving significant progress, with stem cell-derived exosomes emerging as a promising “stem-cell-free” approach for future treatments.

Looking forward, the focus will shift toward combining diverse strategies. The integration of cutting-edge materials, growth factors, and gene therapy holds great potential. Crucially, tailoring these treatments to individual patient needs through personalized approaches will maximize their effectiveness.

## Figures and Tables

**Table 2:** Overview of third-generation membranes: functionality, controlled release, and applications.

Third-Generation Membranes	Functionality	Controlled Release	Applications	References
Polymeric Nanofiber Membranes	These membranes are fabricated using electrospinning techniques, allowing for the integration of growth factors, antibiotics, and other bioactive molecules into the nanofibers.	The fibers can be engineered to degrade at specific rates, releasing incorporated agents in a controlled manner over time. This minimizes the need for high dosages and reduces the potential for side effects, such as systemic toxicity or local irritation.	They are particularly useful in environments where infection control is critical, as they can gradually release antibiotics while simultaneously promoting bone regeneration through growth factors.	[62,63]
Collagen-Based Membranes	Collagen membranes, often combined with other biocompatible materials like polylactic acid (PLA) or bioactive glass, offer a scaffold that supports cell attachment, proliferation, and differentiation.	These membranes can be loaded with growth factors such as bone morphogenetic protein-2 (BMP-2), which are critical for inducing osteogenesis.	The use of such membranes has been shown to enhance bone density and accelerate the healing process, making them ideal for complex bone defects.	[53]
Electrospun Membranes with Multifunctional Coatings	These membranes are typically coated with bioactive molecules like vascular endothelial growth factor (VGF) to enhance angiogenesis, or anti-inflammatory agents to reduce post-surgical inflammation	The electrospinning process allows for the creation of membranes with varying pore sizes and fiber diameters, which can be tailored to optimize cell migration and nutrient diffusion while preventing fibrous tissue infiltration	These membranes are commonly used in dental surgeries, especially for bone grafting around dental implants. They serve as barriers to prevent the invasion of soft tissue into the bone defect site, ensuring that the bone has a clear space to regenerate.	[64,65,66]

**Table 3:** Overview of bone grafting in dentistry: types, procedure, and benefits.

Aspect	Description
Purpose	- Provides support for dental implants - Preserves bone after tooth extraction - Treats bone loss due to periodontal disease
Types of Bone Grafts	**Autograft**: bone from the patient’s own body; high biocompatibility **Advantage**: delivers osteogenic cells and growth factors; low risk of rejection or disease transmission **Disadvantage**: pain, risk of infection at the donor site, limited availability [73,74,75].
	**Allograft**: bone from a human donor; no need for a second surgical site **Advantage**: greater availability, no additional surgery required **Disadvantage**: risk of disease transmission [76,77]
	**Xenograft**: bone from animal sources, usually bovine; effectively stimulates bone growth **Advantage**: widely available, effective in bone regeneration **Disadvantage**: potential for immune reaction and disease transmission [78,79]
	**Alloplast**: synthetic materials; customizable and safe **Advantage**: no risk of disease transmission, customizable to patient needs, can prevent bacterial infections **Disadvantage**: may not integrate as naturally as biological grafts [80,81,82]
Procedure Steps	**Assessment**: imaging to evaluate bone loss **Graft Placement**: insertion of graft material **Healing**: new bone growth and integration **Implant Placement**: implant inserted once sufficient bone density is achieved
Benefits	- Provides a stable foundation for dental implants - Improves aesthetics by maintaining jaw contours - Prevents further bone loss and supports teeth

**Table 4:** Applications, advantages, and challenges of BMPs in alveolar bone regeneration.

I. Applications of BMPs in Alveolar Bone Regeneration
**Boosting Bone Healing:** BMP-2 and BMP-7 are proteins used in medical treatments to promote bone healing, especially in the jaw. BMP-2 is highly effective for quick and strong bone formation, commonly used in dental implants and bone repairs. BMP-7 is used for more complex cases, such as non-healing fractures. **Used in Guided Bone Regeneration (GBR):** BMPs are added to barrier membranes that block soft tissue from entering the bone defect area while gradually releasing BMPs to stimulate bone growth. This combination enhances and regulates bone healing, making it faster and more reliable, especially for implants or trauma repair. **Treating Periodontal Defects:** BMPs are effective for treating severe bone loss from gum disease by regenerating alveolar bone and the periodontal ligament, which stabilizes teeth. They are a reliable option for restoring damaged areas to support teeth or prepare for implants when other treatments fall short. **Sinus Augmentation:** BMPs are used in sinus lifts to promote bone growth in the upper jaw when there is not enough bone to secure dental implants. They help build a strong foundation for the implant and speed up healing, increasing the procedure’s success rate.
**II. Advantages of Using BMPs**
**Accelerated Healing:** BMPs significantly reduce the healing time by accelerating bone formation at the graft site. **Improved Outcomes**: The use of BMPs leads to more predictable and successful outcomes in both bone grafting and GBR procedures. **Reduced Need for Secondary Procedures**: By enhancing bone regeneration, BMPs reduce the need for secondary bone grafting procedures, making the overall treatment process more efficient.
**III. Challenges and Considerations**
**Cost:** One downside is that BMPs can be pretty pricey, which can limit their use in routine dental or bone procedures. Not every patient can afford them. **Side Effects:** There is always a chance of side effects with BMPs, like bone forming in places where it should not (ectopic bone formation) or causing inflammation. These are risks doctors need to consider carefully. **Dosage and Delivery:** The correct dosage and delivery method are critical to the success of BMP therapy. Overuse or improper application can lead to complications such as excessive bone growth or unwanted tissue reactions.

**Table 5:** Stem cell-based regenerative strategies for bone and dental tissue.

Stem Cell-Based Regenerative Strategies	Mechanism	Examples	References
Regeneration with Endogenous Stem Cells	- Activation of resident stem cells; e.g., bone marrow mesenchymal stem cells (BMMSCs), dental pulp stem cells (DPSCs), periodontal ligament stem cells (PDLSCs). - Use of signaling molecules (e.g., growth factors, cytokines) to stimulate the body’s stem cells. - Scaffold-guided tissue regeneration, where scaffolds are used to support the migration and differentiation of endogenous cells.	- Bone regeneration through endogenous BMMSCs responding to injury. - Tooth pulp and periodontal ligament (PDL) regeneration using dental pulp stem cells (DPSCs) and periodontal ligament stem cells (PDLSCs) in situ.	[111]
Regeneration with Exogenous Stem Cells	- Using external cell sources: bone marrow mesenchymal stem cells (BMMSCs), dental pulp stem cells (DPSCs), periodontal ligament stem cells (PDLSCs), adipose-derived mesenchymal stem cells (ADMSCs). - Transplantation of stem cells to the injury site. - Tissue engineering constructs using stem cells, scaffolds, and growth factors to create bioengineered grafts. - Systemic administration: stem cells delivered via bloodstream for targeting damaged tissues.	- Bone grafts using exogenous bone marrow mesenchymal stem cells (BMMSCs), or adipose-derived mesenchymal stem cells (ADMSCs) to regenerate large bone defects. - Dental pulp regeneration using SHED or DPSCs in root canals.	[92,112,113,114,115,116]
